# Genetic associations link lung cancer liability to brain imaging phenotypes and nominate an FN1-associated tumor–oligodendrocyte program in small-cell lung cancer

**DOI:** 10.3389/fonc.2026.1890325

**Published:** 2026-09-09

**Authors:** Kai Xu, Peihang Xu, Kunsong Su, Zhenkun Liu, Yang Hao, Hongxiang Feng, Guowei Che, Qinghua Zhou, Xiaoqian Zhai

**Affiliations:** 1 Department of Thoracic Surgery, West China Hospital, Sichuan University, Chengdu, China; 2 Department of Thoracic Surgery, China Japan Friendship Hospital, Beijing, China; 3 Lung Cancer Center, West China Hospital, Sichuan University, Chengdu, Sichuan, China; 4 Department of Medical Oncology, Cancer Centre, West China Hospital, Sichuan University, Chengdu, Sichuan, China

**Keywords:** brain metastasis, Mendelian randomization, oligodendrocyte, single-cell RNA sequencing, small-cell lung cancer, white matter remodeling

## Abstract

**Background:**

Brain metastasis is a major cause of mortality in lung cancer, particularly in small-cell lung cancer (SCLC). Whether genetic susceptibility to lung cancer is associated with brain structural and functional traits relevant to metastatic vulnerability remains unclear. We investigated subtype-specific genetic associations with brain imaging phenotypes and explored the biological context of the SCLC-associated white-matter signal.

**Methods:**

Bidirectional two-sample Mendelian randomization (MR) was performed using European genome-wide association study summary statistics for three lung cancer subtypes, 3,935 structural MRI phenotypes, and 191 resting-state functional MRI traits. MR findings were evaluated using genetic-correlation and sensitivity analyses. Public single-cell and spatial transcriptomic datasets from brain metastases, computational ligand-receptor inference, and virtual perturbation modeling were used to explore the SCLC-related findings.

**Results:**

SCLC genetic liability showed the clearest white-matter-related pattern, involving the superior cerebellar peduncle and cerebellar-related functional connectivity, whereas lung squamous cell carcinoma was mainly associated with frontal and parietal traits. Single-cell analysis identified mature-like, intermediate, and reactive-like oligodendrocyte states. Reactive-like cells exhibited reduced myelination-associated genes and enhanced stress-response programs. Computational analyses prioritized an FN1-associated tumor-oligodendrocyte interaction program, with neuronal-like malignant SCLC cells as the major predicted sender population. Cross-dataset, spatial, and virtual perturbation analyses provided additional exploratory support for tumor-derived FN1 as a candidate niche-organizing signal associated with reactive oligodendrocyte remodeling.

**Conclusions:**

Lung cancer subtypes show distinct genetic associations with brain phenotypes. The SCLC findings and exploratory multi-omics analyses support an FN1-centered tumor-oligodendrocyte model that warrants experimental validation.

## Introduction

Lung cancer is the leading cause of cancer-related mortality worldwide (1, 2). It is broadly classified as non-small-cell lung cancer (NSCLC), which mainly includes adenocarcinoma and squamous cell carcinoma, or small-cell lung cancer (SCLC) (3). Outcomes remain poor in advanced disease: the 5-year survival rate for advanced NSCLC is approximately 5%-10% (4), and two-thirds of patients with SCLC have extensive-stage disease at diagnosis, with median overall survival rarely exceeding 15 months (5–7).

Metastasis is the principal cause of cancer-related death (8). Lung cancer accounts for approximately half of all brain metastases and affected patients have a median overall survival of about 7 months (9–12). The burden is particularly high in SCLC, in which up to 75% of patients may ultimately develop brain metastases (6, 13). Brain metastasis therefore displays marked organ selectivity (14). Two major hypotheses have been proposed to explain organ-specific metastasis (15). The hemodynamic or mechanical hypothesis (15) emphasizes anatomical routes of dissemination, such as the Batson venous plexus connecting breast and pelvic tumors with the brain (16). In lung cancer, however, pulmonary venous drainage alone does not fully explain the marked brain tropism. The seed-and-soil hypothesis (17) proposes that disseminated tumor cells form secondary tumors only in permissive organ-specific microenvironments (18) and is widely accepted in the context of lung cancer brain metastasis (15). GWAS have identified new lung cancer susceptibility loci and histology-dependent genetic heterogeneity (19–21). Because lung cancer heritability is approximately 18%, McKay et al. identified 18 additional susceptibility loci (19). Studies by Shi et al. and Gorman et al. further revealed ancestry-related differences in genetic susceptibility (20, 21). The widely used TRICL consortium datasets (22, 23) have provided additional insight into the genetic architecture of lung cancer, raising the possibility that inherited susceptibility may also be associated with brain phenotypes relevant to metastatic vulnerability.

**Figure 1:**
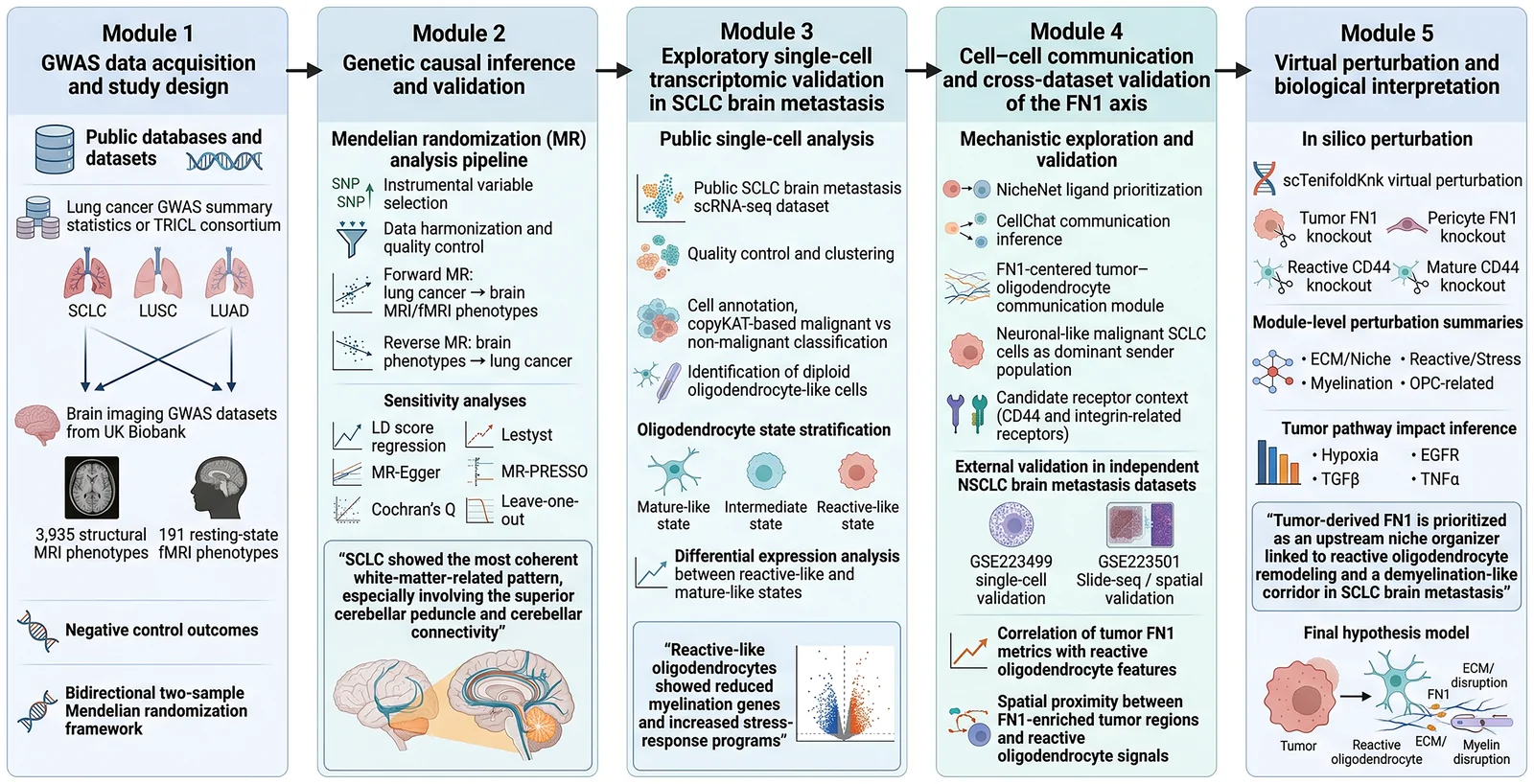
Overall study workflow. Public GWAS summary statistics for SCLC, LUSC, and LUAD; 3,935 structural MRI phenotypes; and 191 resting-state fMRI phenotypes were analyzed using bidirectional two-sample MR. Instrument selection, harmonization, quality control, genetic-correlation assessment, and sensitivity analyses were used to identify subtype-specific genetic associations with brain phenotypes. The coherent SCLC white-matter pattern motivated exploratory single-cell analysis of oligodendrocyte states and computational inference of tumor-oligodendrocyte interactions. Independent NSCLC brain-metastasis single-cell and spatial datasets were used for supportive cross-dataset evaluation, and scTenifoldKnk virtual perturbation was used to prioritize candidate niche-associated regulatory signals. The downstream analyses were hypothesis generating and did not constitute experimental validation.

Identifying patients at high risk of brain metastasis is important for clinical management (24). MRI provides quantitative measures of brain structure and function and is routinely used to evaluate patients with suspected brain metastasis. Blood-brain barrier disruption and angiogenesis are involved in brain metastatic progression (25, 26), and related vascular changes may be detectable by MRI (26). Regional structural alterations have also been associated with brain metastasis (27–29). Imaging-genetics studies have subsequently linked common genetic variation to brain MRI phenotypes (30). Elliott et al. performed a structural MRI GWAS in 39,691 UK Biobank participants (31), a large prospective population resource (32), whereas Zhao et al. mapped genetic variation associated with functional MRI traits (33). Resting-state fMRI measures spontaneous brain function through blood-oxygen-level-dependent contrast (34, 35), which may also reflect regional vascular changes (36). MR uses genetic variants as instrumental variables to reduce conventional confounding and reverse causation (37), but its estimates reflect genetically predicted liability rather than direct remodeling during tumor progression. We therefore evaluated three lung cancer subtypes against 3,935 structural MRI phenotypes (31) and 191 functional MRI phenotypes (33, 38). We hypothesized that susceptibility to different lung cancer subtypes may be associated with distinct brain imaging traits and that the SCLC-related white-matter pattern may provide clues to cellular programs relevant to brain metastatic vulnerability. Because oligodendrocytes are the principal regulators of myelination, axonal insulation, and white-matter homeostasis, they were prioritized for downstream exploratory analysis (39). Nevertheless, astrocytes, microglia, endothelial cells, and extracellular-matrix components also contribute to white-matter biology and remain plausible alternative or complementary explanations (40). Genetic-correlation estimates, sensitivity analyses, and reverse MR were used to assess the robustness of the genetic findings (**Figure 1**).

We further investigated whether the tumor-side signal associated with oligodendrocyte-state variation was more consistent with a direct ligand effect or with a broader niche-organizing cue. FN1 emerged as a plausible candidate because it was retained within the displayed high-priority ligand set, was broadly expressed across malignant SCLC states, and was repeatedly represented in extracellular-matrix interaction programs. We therefore evaluated a conservative, hypothesis-generating model in which high-tail tumor FN1 is associated with reactive-like oligodendrocyte features, shows regional spatial proximity to reactive oligodendrocyte signals, and produces coherent predicted network changes after virtual perturbation. This framework was intended to evaluate biological plausibility rather than establish a definitive linear mechanism.

## Methods

### GWAS of lung cancer

GWAS summary statistics for lung cancer were obtained from the TRICL consortium and included three pathological subtypes: LUAD (11,245 cases and 54,619 controls), LUSC (7,704 cases and 54,763 controls), and SCLC (2,791 cases and 20,580 controls) (23). The details of lung cancer datasets were shown in **Supplementary Table 1**.

### GWAS of brain structural MRI

The structural brain MRI corresponding genetic variants were obtained from the Elliott’s GWAS analysis study based on UKBB (31). In our study, all 3935 brain structural phenotypes were employed to investigate the associations between different lung cancer and brain structural.

### GWAS of brain functional MRI

Genetic associations for resting-state fMRI phenotypes were obtained from the UK Biobank-based GWAS reported by Zhao et al. (33, 38, 41). We analyzed 191 retained rs-fMRI traits, including node amplitude measures, pairwise functional connectivities, and global connectivity traits. Details of these phenotypes are summarized in **Supplementary Table 2**.

### GWAS of negative control

Negative-control analyses were performed using height (42) and birth length (43) as outcomes to evaluate the suitability of the selected lung cancer instruments. The corresponding results are presented in **Supplementary Table 9**.

### Instruments selection

Instrumental variables were selected according to standard MR assumptions (44). SNPs associated with the exposure at genome-wide significance (P < 5 x 10^-8) were retained and pruned for linkage disequilibrium at r2 < 0.001 within a 10,000-kb window using the European 1000 Genomes reference panel. Potential variants associated with major confounders, including education and socioeconomic status, were evaluated using PhenoScanner and excluded when appropriate (45–48).

### Data harmonization

Exposure and outcome datasets were harmonized using the harmonise_data function in TwoSampleMR (version 0.6.3). Effect alleles were aligned, and ambiguous palindromic variants were excluded. Variants directly associated with the outcome at genome-wide significance (P < 5 x 10^^-8^) were removed to reduce potential violation of the exclusion-restriction assumption.

### Quality control of instrumental variables

Instrument strength and directionality were evaluated before MR analysis. Weak instruments with F-statistics < 10 were excluded (49), and Steiger filtering was used to remove variants for which the inferred direction from exposure to outcome was unsupported.

### Bidirectional two-sample mendelian randomization analyses

Bidirectional two-sample MR was used to evaluate potential causal relationships between genetically predicted lung cancer liability and brain structural or functional phenotypes. In forward analyses, lung cancer was modeled as the exposure and MRI phenotypes as outcomes; in reverse analyses, MRI phenotypes were modeled as exposures and lung cancer as the outcome. The Wald ratio was used for single-SNP instruments, whereas inverse variance weighting (IVW) was the primary method for multi-SNP models (48). Structural MRI associations with FDR-adjusted P < 0.05 were considered statistically significant. Because rs-fMRI traits are high dimensional and sensitive to physiological and technical variation, nominal P < 0.01 was prespecified only as an exploratory threshold; these functional findings were classified as suggestive rather than confirmatory (31, 41). Results were visualized using ggseg, ggseg3d, and BrainNet Viewer (50, 51). MR estimates were interpreted as effects of genetic liability under the instrumental-variable assumptions and not as direct evidence of tumor-induced remodeling. No confirmatory conclusion was based solely on nominal rs-fMRI associations, which were retained only for descriptive hypothesis generation and were not multiplicity confirmed.

### Evaluation of genetic correlations

For statistically significant forward MR associations, shared genetic architecture was evaluated using LD score regression (LDSC) as a complementary analysis (52, 53). LDSC estimates genome-wide genetic correlation and therefore addresses a different question from instrument-based MR (53, 54). LD scores were calculated using the 1000 Genomes European reference data, and variants with minor allele frequency < 0.01 or imputation INFO score < 0.9 were excluded (55).

### Sensitivity analyses

Sensitivity analyses were performed for exposure–outcome pairs with more than three instruments. These analyses included leave-one-out testing, MR-PRESSO global testing, MR-Egger intercept testing, and Cochran’s Q heterogeneity testing (56, 57).

### Exploratory public single-cell transcriptomic analysis in SCLC brain metastasis

To explore cellular programs potentially related to the SCLC-associated white-matter phenotypes, we analyzed public single-cell transcriptomic data from SCLC brain metastases. After standard quality control, low-quality cells and potential doublets were removed, and preprocessing, clustering, and visualization were performed in Seurat (58). A conservative singlet-retention strategy was used to reduce instability from single-algorithm doublet calling. Oligodendrocyte biology was prioritized because of its central role in myelin maintenance and white-matter homeostasis; other brain-resident populations were retained for comparative annotation but were not the primary focus of the downstream hypothesis-generating analyses.

Cell-type annotation integrated reference-based labeling, canonical marker genes, and copy-number-based malignant-cell inference. Preliminary annotation was performed with SingleR and celldex, and large-scale copy-number patterns were inferred with copyKAT to distinguish aneuploid malignant cells from diploid non-malignant cells (59, 60). Diploid oligodendrocyte-like cells were then identified using established oligodendrocyte and myelination-associated markers, including MOG, MAG, PLP1, MBP, and MOBP.

Oligodendrocyte heterogeneity was characterized by module scoring of predefined mature/myelinating, oligodendrocyte precursor-like, and reactive/stress-associated programs. On the basis of relative module enrichment, oligodendrocyte-like cells were stratified into mature-like, intermediate, and reactive-like states, and differential expression between reactive-like and mature-like cells was performed with FindMarkers in Seurat.

Potential tumor-oligodendrocyte signaling programs were explored with two complementary computational frameworks. NicheNet was applied to prioritize candidate tumor-derived ligands associated with the reactive-like oligodendrocyte transcriptional program. CellChat was used to infer potential extracellular-matrix-receptor and secreted ligand-receptor interaction patterns after updating oligodendrocyte subgroup annotations. Receiver-side expression of candidate receptors was examined across oligodendrocyte states. These predictions were interpreted as candidate interactions and not as experimentally confirmed cell-cell communication.

### Cross-dataset evaluation of the FN1-reactive oligodendrocyte association and virtual perturbation analyses

To evaluate whether FN1 was more consistent with a direct tumor-directed ligand or a broader niche-associated signal, we performed supportive analyses in independent NSCLC brain metastasis datasets. In GSE223499, broad cell-type annotation and oligodendrocyte-state scoring were used to derive sample-level tumor FN1 metrics, including mean expression, upper-tail q90 expression, and the fraction of FN1-high tumor cells. Spearman correlations were calculated between these tumor metrics and reactive-state features, including mean reactive score and reactive-like cell fraction. Because a localized extracellular-matrix sender signal may be better captured by upper-tail than by global mean expression, q90 FN1 and the FN1-high tumor fraction were prespecified as the principal exploratory metrics.

For spatial evaluation, Slide-seq data from GSE223501 were reconstructed and integrated with the reference single-cell atlas using CellTrek. Spatial association between FN1-enriched tumor regions and reactive-like oligodendrocyte signals was evaluated by comparing observed tumor-to-reactive proximity with permutation-based null distributions and by inspecting representative sections and regions of interest. Spatial proximity was interpreted as regional co-localization rather than direct molecular interaction. Finally, scTenifoldKnk was used to prioritize candidate regulatory genes through in silico perturbation modeling, including FN1 knockout in tumor-like and pericyte-like populations and CD44 knockout in reactive-like and mature-like oligodendrocytes. Significantly perturbed genes were summarized using predefined ECM/Niche, Reactive/Stress, Myelination, and OPC-related modules, and enrichment was assessed using Fisher’s exact test. Virtual perturbation effects were considered hypothesis-generating predictions rather than experimentally validated knockout outcomes.

## Results

### MR identifies subtype-specific genetic associations with brain imaging phenotypes

**Figure 2:**
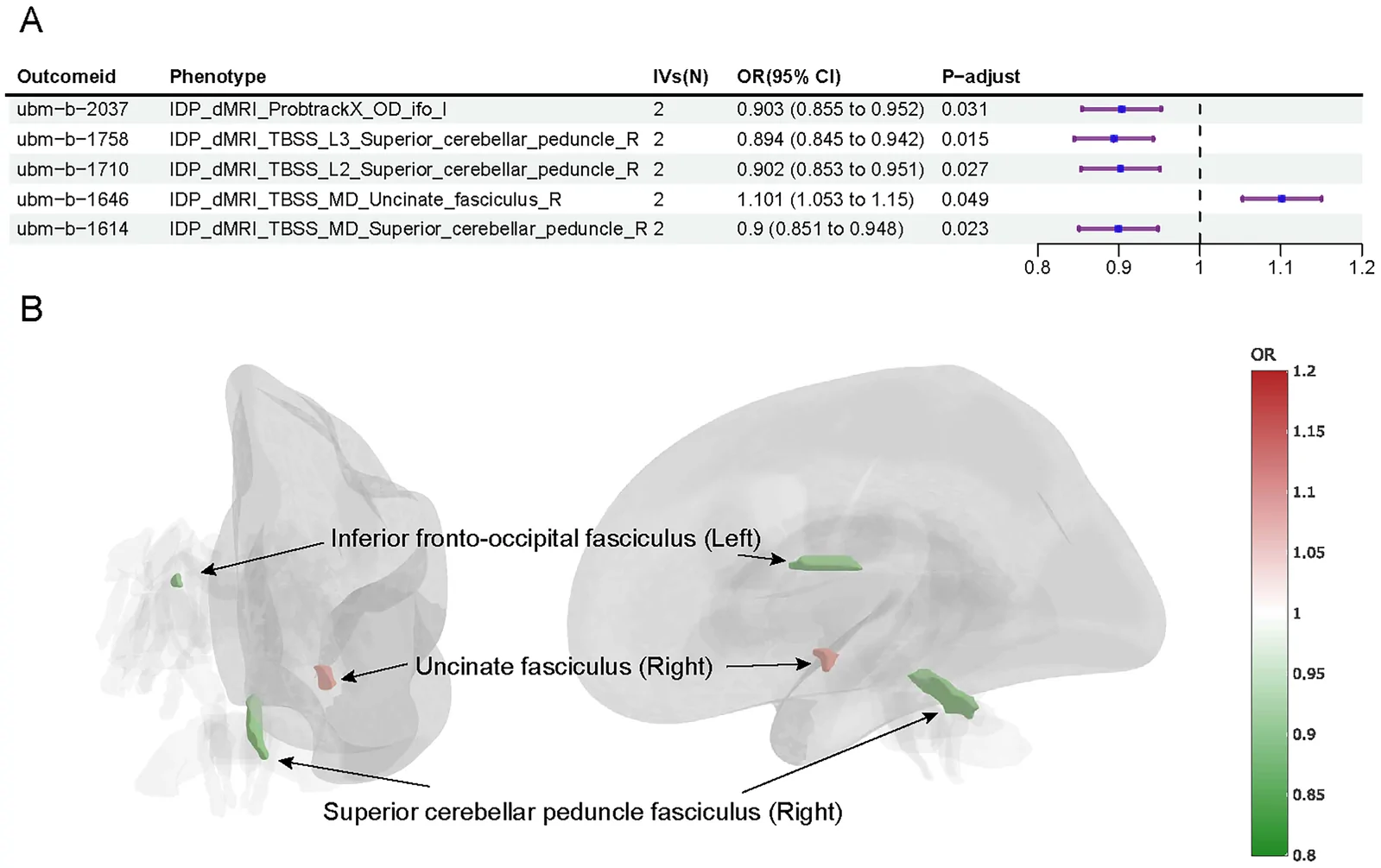
MR associations of SCLC liability with structural MRI phenotypes. The SCLC GWAS included 2,791 cases and 20,580 controls. **(A)** Forest plot of statistically significant IVW associations after FDR correction (adjusted P < 0.05). ProbtrackX denotes tractography-derived inferior fronto-occipital fasciculus measures. Effect estimates and 95% confidence intervals represent the change in each standardized MRI phenotype associated with genetically predicted SCLC liability. **(B)** Anatomical representation of the corresponding white-matter tracts; color denotes effect size. MR results reflect genetic-liability associations under the instrumental-variable assumptions rather than direct tumor-induced structural remodeling.

**Figure 3:**
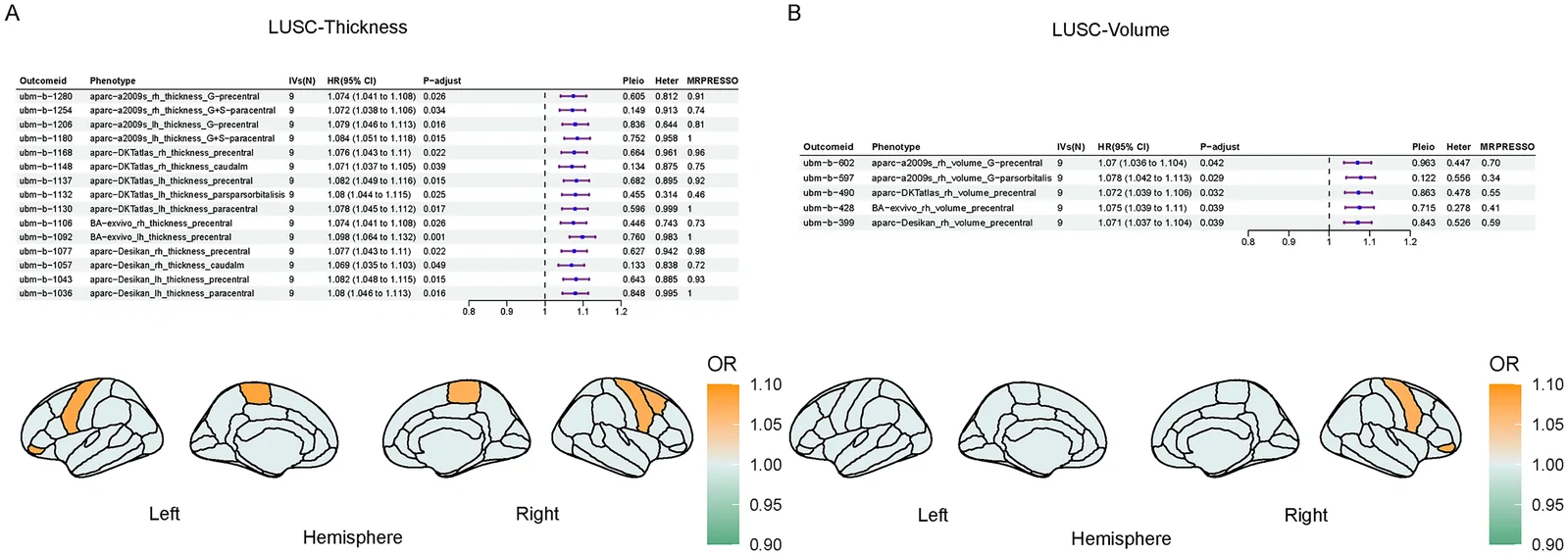
MR associations of LUSC liability with structural MRI phenotypes. The LUSC GWAS included 7,704 cases and 54,763 controls. Upper panels show statistically significant IVW associations after FDR correction (adjusted P < 0.05), with effect estimates and 95% confidence intervals for standardized MRI phenotypes. Heter, Pleio, and MRPRESSO indicate the P values from Cochran’s Q heterogeneity, MR-Egger intercept, and MR-PRESSO global tests, respectively. Lower panels show anatomical maps of corresponding regions; when several associations mapped to one region, the mean effect was displayed. **(A)** Cortical thickness associations. **(B)** Brain-volume associations.

MR analyses identified distinct subtype-specific genetic associations with brain structural and functional phenotypes. SCLC liability showed the most coherent white-matter-related pattern, with associations involving the superior cerebellar peduncle and limited suggestive cerebellar-related functional connectivity changes (**Figure 2**, **Supplementary Figure 1**, **Supplementary Tables 3**–**S6**). LUSC was mainly associated with cortical structural traits and exploratory functional alterations in frontal, parietal, and sensorimotor regions (**Figure 3**, **Supplementary Figure 2**, **Supplementary Tables 3**-**S6**). LUAD showed no statistically significant structural associations and only limited exploratory functional signals without supporting genetic correlations (**Supplementary Figure 3**, **Supplementary Tables 5**, **S6**). Reverse MR yielded several exploratory brain-to-lung associations that were interpreted cautiously (**Supplementary Figure 4**, **Supplementary Tables 7**, **S8**). These findings identify associations between inherited susceptibility and imaging traits rather than direct evidence of tumor-induced brain remodeling. The coherent SCLC white-matter pattern motivated the subsequent hypothesis-generating cellular analyses.

### Exploratory single-cell analysis characterizes oligodendrocyte states and predicted FN1-associated tumor-oligodendrocyte interactions in SCLC brain metastasis

**Figure 4:**
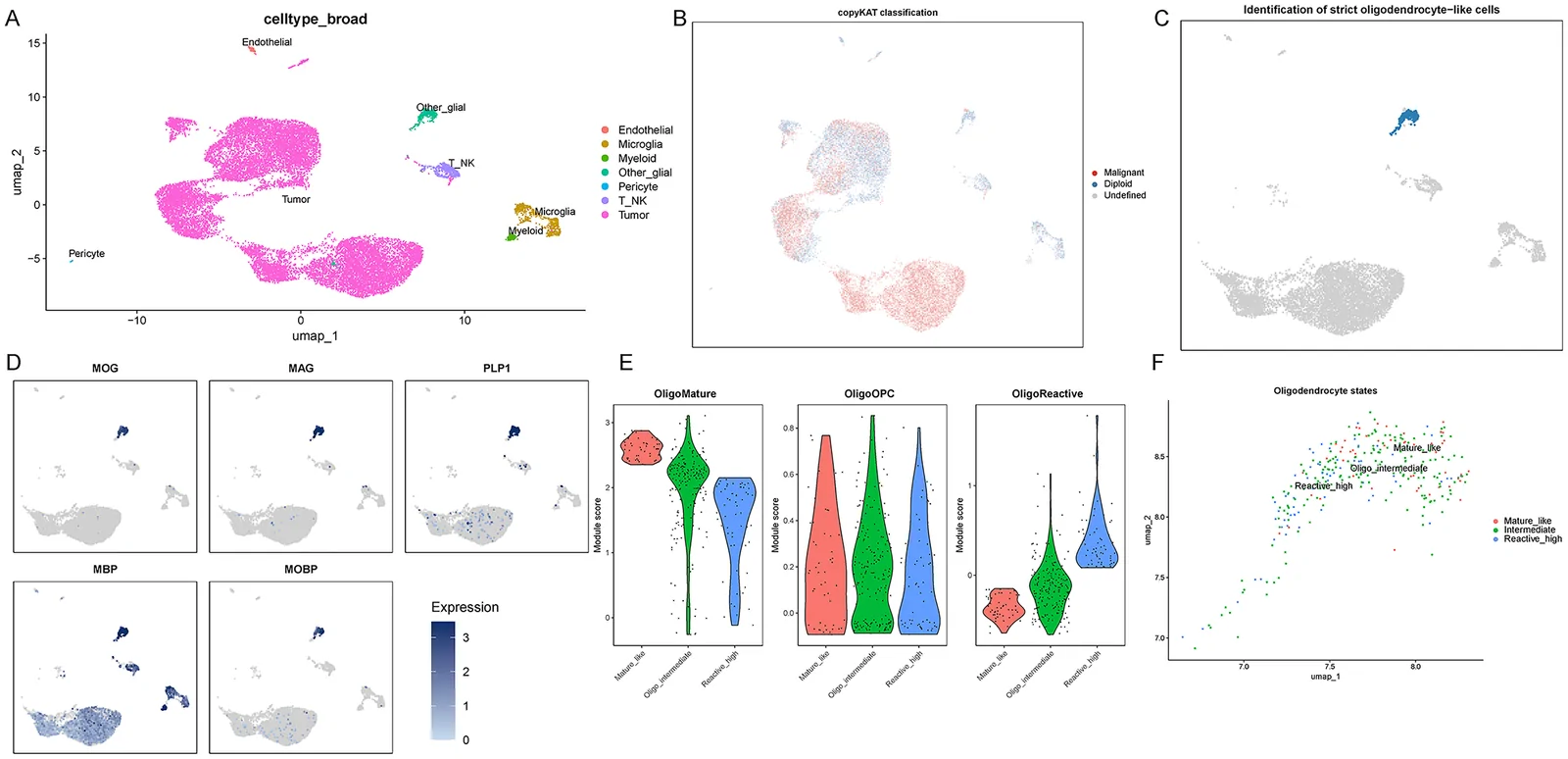
Exploratory single-cell characterization of oligodendrocyte-associated states in SCLC brain metastasis. **(A)** UMAP of the public SCLC brain-metastasis single-cell dataset after quality control, clustering, and cell-type annotation. **(B)** copyKAT inference of large-scale copy-number patterns distinguishing aneuploid malignant cells from diploid non-malignant cells; unstable classifications are shown as undefined. **(C)** UMAP highlighting the strict oligodendrocyte-like population, defined as diploid cells within the oligodendrocyte-like cluster on the basis of integrated annotation, canonical markers, and copy-number inference. **(D)** Feature plots of MOG, PLP1, MBP, and MOBP. **(E)** Violin plots of mature/myelinating, precursor-like, and reactive/stress module scores across oligodendrocyte subgroups. **(F)** UMAP of Mature_like, Oligo_intermediate, and Reactive_high states. The analysis was based on one SCLC brain-metastasis specimen (GSM8842608).

To provide cellular context for the SCLC-associated white-matter imaging pattern, we analyzed public single-cell transcriptomic data from SCLC brain metastases. After quality control, dimensionality reduction, and cell annotation, copy-number inference distinguished high-confidence aneuploid malignant SCLC cells from diploid non-malignant microenvironmental cells. Within the diploid compartment, we identified oligodendrocyte-like cells expressing canonical oligodendrocyte and myelination markers, including MOG, MAG, PLP1, MBP, and MOBP (**Figures 4A–D**). This analysis did not directly connect the MR associations to a cellular mediator; rather, it identified a biologically relevant white-matter cell population for downstream exploration.

**Figure 5:**
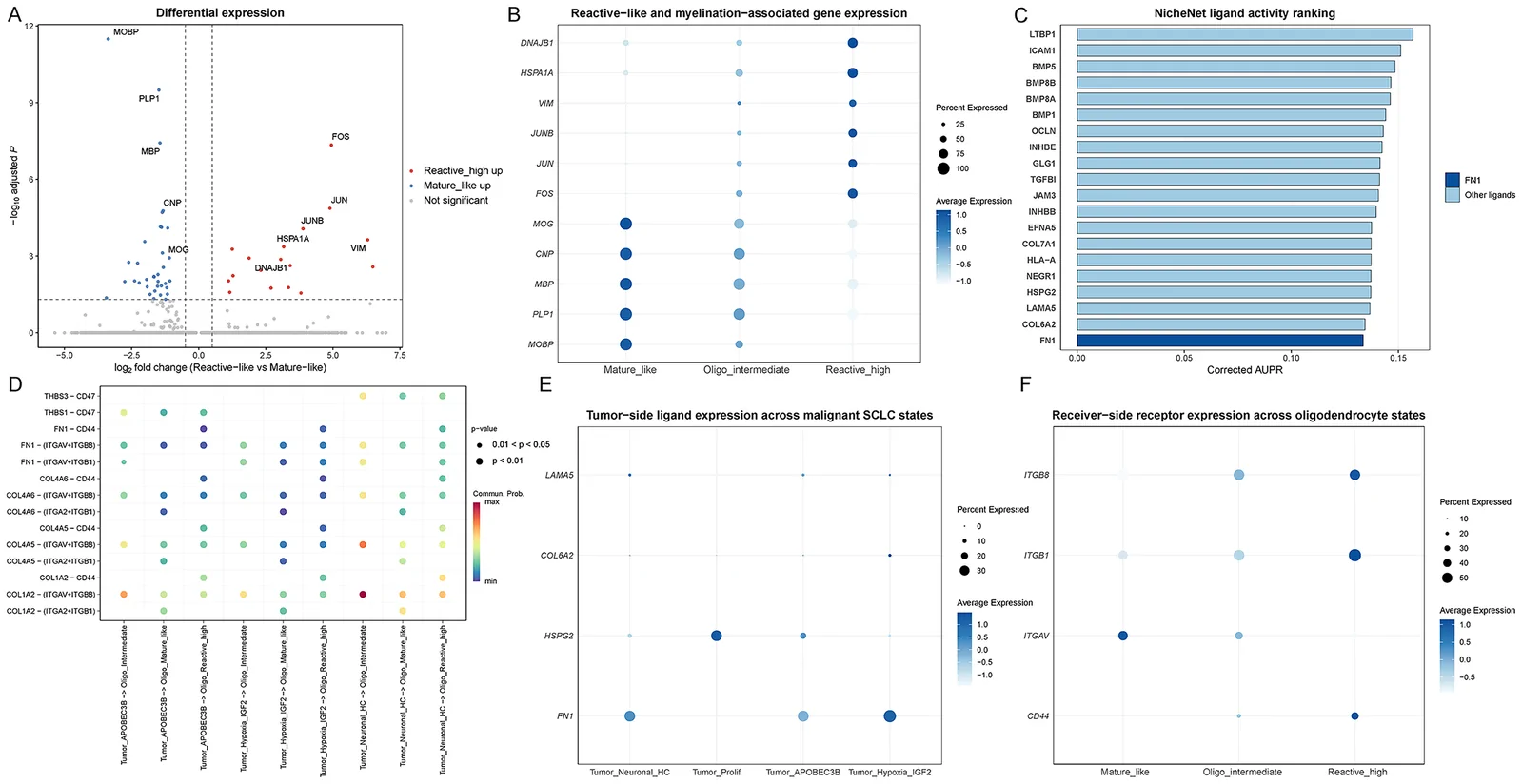
Oligodendrocyte transcriptional states and computationally inferred FN1-associated tumor-oligodendrocyte interactions in SCLC brain metastasis. **(A)** Differential expression between reactive-like and mature-like oligodendrocytes. Genes enriched in reactive-like cells are shown on the right and genes enriched in mature-like cells on the left; adjusted P values were calculated from the Seurat Wilcoxon rank-sum test. **(B)** Dot plot of reactive/stress and myelination-associated genes; dot size indicates the expressing-cell fraction and color indicates average expression. **(C)** NicheNet ligand-activity ranking of candidate tumor-derived ligands associated with the reactive-like transcriptional program; FN1 is highlighted as a convergent candidate selected from the displayed high-priority ligand set. **(D)** CellChat summary of predicted tumor-to-oligodendrocyte interactions. Bubble size indicates the number of inferred interactions and color indicates summed predicted communication probability. **(E)** Tumor-side ligand expression across malignant SCLC states. **(F)** Receiver-side receptor expression across oligodendrocyte states. These ligand-receptor results are computational predictions and do not demonstrate direct cell-cell communication. **(A–F)** were generated from the same SCLC brain-metastasis specimen (GSM8842608).

Module-score-based stratification resolved mature-like, intermediate, and reactive-like oligodendrocyte states (**Figures 4E, F**). Differential expression between reactive-like and mature-like cells showed a clear transcriptional contrast. Reactive-like oligodendrocytes upregulated stress-response and immediate-early genes, including FOS, JUN, JUNB, VIM, HSPA1A, and DNAJB1, whereas mature-like oligodendrocytes retained canonical myelination-associated genes such as MOBP, PLP1, MBP, CNP, and MOG (**Figures 5A, B**). These findings indicate a reactive transcriptional state characterized by attenuation of myelination-related programs rather than establish histological demyelination.

We next computationally evaluated whether malignant SCLC cells could provide candidate signals associated with oligodendrocyte-state variation. NicheNet identified FN1 among the displayed high-priority tumor-derived ligands linked to the reactive-like transcriptional program, while CellChat inferred neuronal-like malignant SCLC cells as the major predicted sender population (**Figures 5C, D**). FN1 was prioritized as a convergent candidate because it was included among the high-priority NicheNet ligands, was broadly expressed across malignant SCLC states, was represented in extracellular-matrix interaction programs, and received additional support from the external spatial and virtual perturbation analyses. Inferred extracellular-matrix-receptor signaling toward intermediate and reactive-like oligodendrocyte states included FN1-CD44 and FN1-associated integrin-containing receptor complexes. These analyses nominate candidate interactions but do not demonstrate direct signaling.

Receiver-side expression provided a permissive context for these predictions. Oligodendrocyte-like cells broadly expressed integrin-related receptors, including ITGAV, ITGB1, and ITGB8. CD44 was more enriched in reactive-like than in mature-like oligodendrocytes, suggesting that FN1-CD44 may be relatively reactive-state biased, whereas FN1-integrin interactions may represent a broader extracellular-matrix context (**Figures 5E, F**). Together, the single-cell analyses support a hypothesis in which SCLC-associated white-matter imaging traits are biologically contextualized by oligodendrocyte-state heterogeneity and a predicted FN1-associated tumor-oligodendrocyte interaction program.

### Cross-dataset evaluation and virtual perturbation provide exploratory support for an indirect FN1-reactive oligodendrocyte model

**Figure 6:**
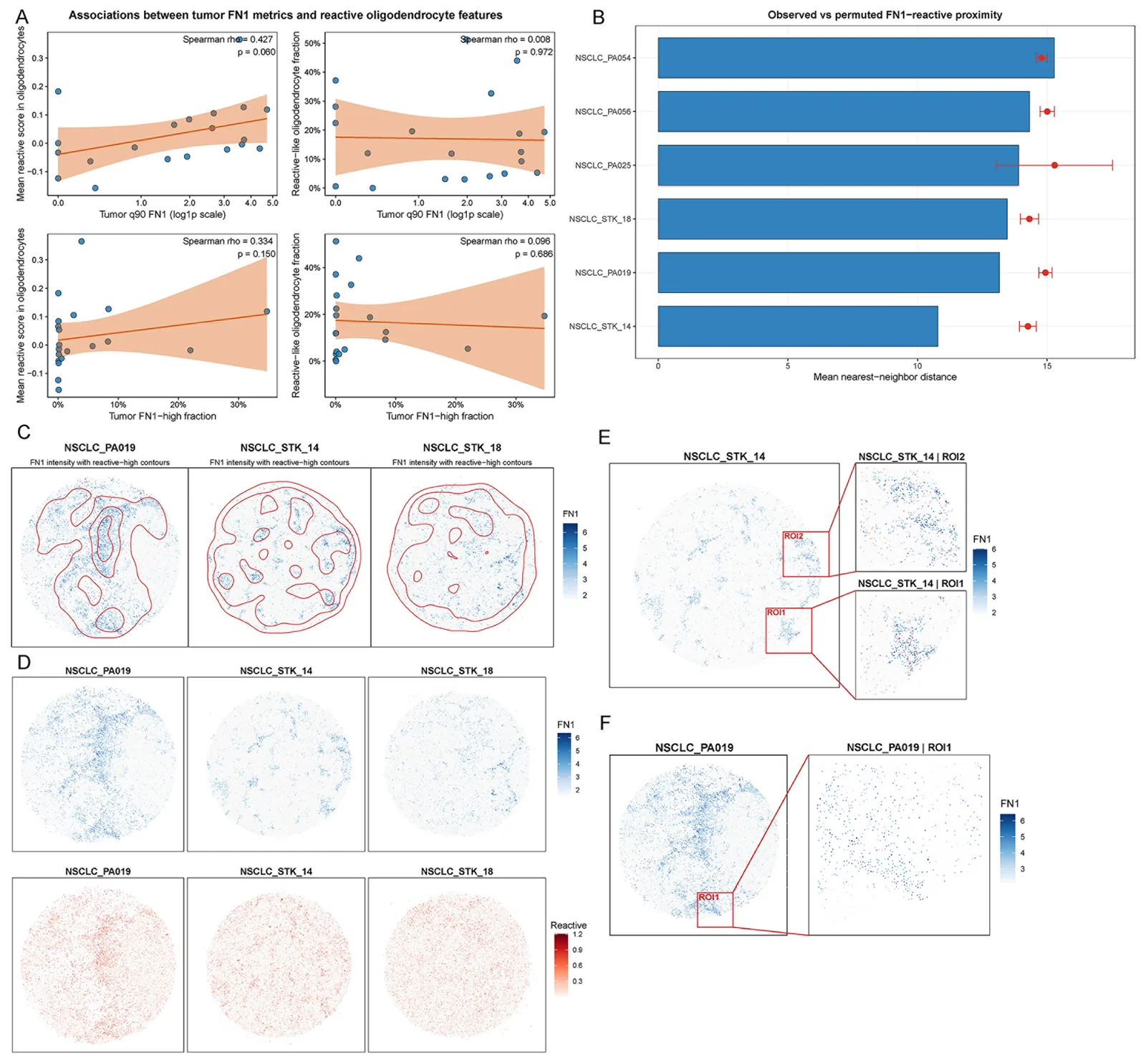
Cross-dataset evaluation of the FN1-reactive oligodendrocyte association. **(A)** Sample-level associations between tumor FN1 metrics and reactive oligodendrocyte features in GSE223499. Spearman rho and two-sided P values are displayed in each panel; the number of evaluable samples is represented by the plotted points. **(B)** Observed versus permuted FN1-reactive proximity in evaluable GSE223501 spatial samples. Blue bars show observed mean nearest-neighbor distances; red points and horizontal intervals show permutation-based expectations. **(C)** Representative section-level FN1 intensity maps with high-intensity contours. **(D)** Representative FN1 and reactive oligodendrocyte signal maps. **(E, F)** Region-of-interest views from NSCLC_STK_14 and NSCLC_PA019. These analyses were exploratory; spatial proximity indicates regional co-localization rather than direct signaling. **(A)** included 20 evaluable brain-metastasis samples. **(B)** included six spatial samples, with 200 label permutations per sample; **(C–F)** show representative sections or regions of interest.

Because direct sender-to-tumor NicheNet support for FN1 was weak, we evaluated a more conservative indirect model in independent NSCLC brain metastasis data. In GSE223499, mean tumor FN1 showed only modest associations with oligodendrocyte reactive-state features, whereas upper-tail tumor FN1 better captured the putative localized sender signal. Tumor q90 FN1 showed a borderline positive association with mean oligodendrocyte reactive score (Spearman rho approximately 0.427, P approximately 0.060), while associations with reactive-like cell fraction were weak or absent (**Figure 6A**). These results were treated as exploratory trends rather than statistically robust validation. GSE223499 also recapitulated mature-like, reactive-like, and OPC-like oligodendrocyte states and an extracellular-matrix-associated expression background (**Supplementary Figure 9**).

Spatial analyses in GSE223501 provided additional exploratory evidence. Across evaluable Slide-seq samples, observed FN1-reactive proximity tended to be shorter than permutation-based expectations, and representative sections showed FN1-enriched tumor territories within or adjacent to reactive-like oligodendrocyte-enriched regions (**Figures 6B–F**). These observations support localized regional co-occurrence but do not establish direct cellular signaling or functional dependence. The pattern was more consistent with a localized high-tail FN1 signal than with globally increased FN1 abundance.

**Figure 7:**
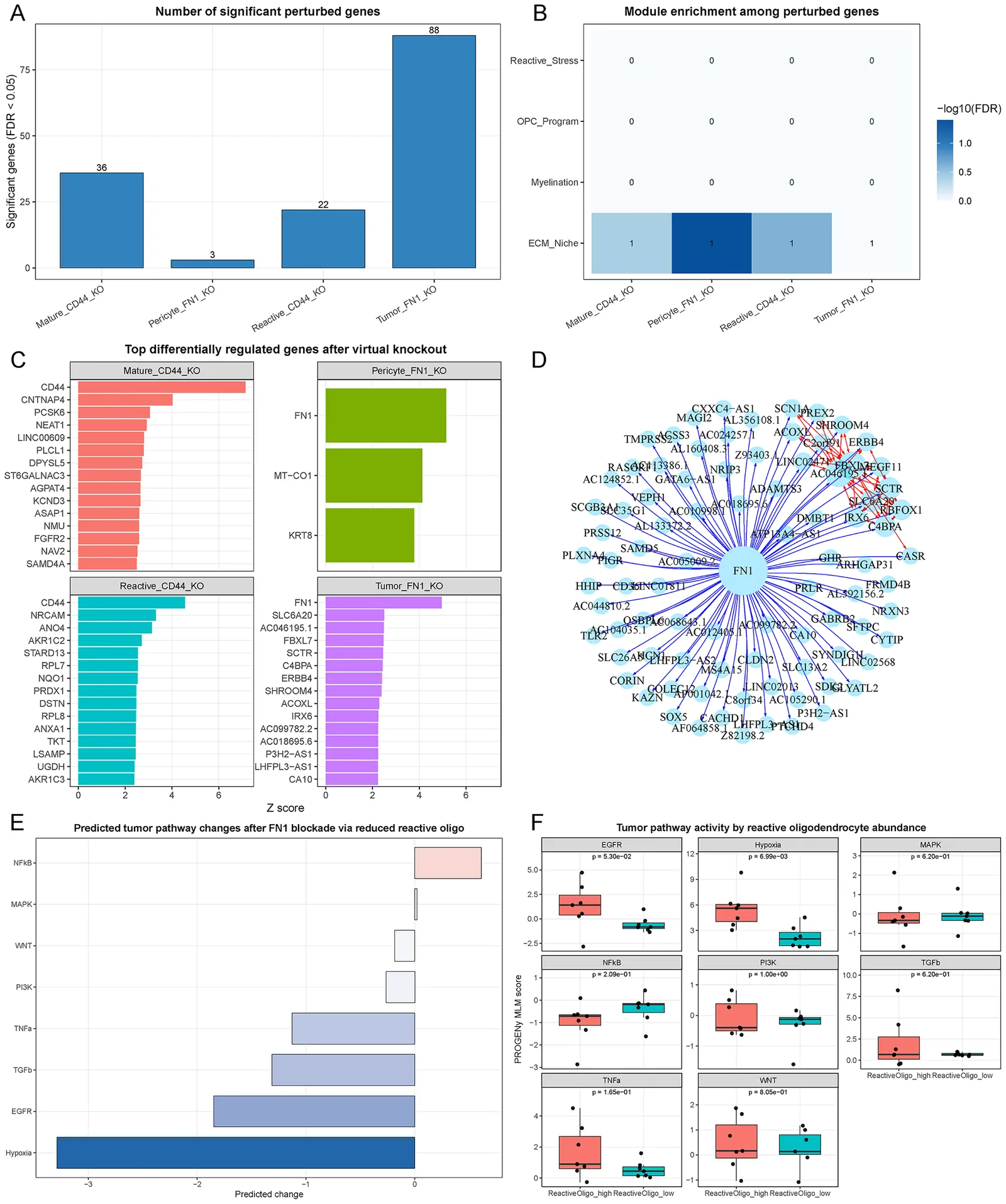
Virtual perturbation prioritizes tumor-derived FN1 as a candidate niche-associated regulator and links reactive oligodendrocyte abundance to tumor pathway activity. **(A)** Numbers of significantly perturbed genes (FDR < 0.05) after Mature_CD44_KO, Pericyte_FN1_KO, Reactive_CD44_KO, and Tumor_FN1_KO virtual perturbations. **(B)** Enrichment of predefined modules among perturbed genes; color denotes -log10(FDR) from Fisher’s exact test and numbers denote gene overlap. **(C)** Top predicted differentially regulated genes; bar length denotes perturbation-derived Z score. **(D)** FN1-centered network predicted after virtual tumor FN1 knockout. **(E)** Predicted tumor-pathway changes under the indirect model of reduced reactive oligodendrocyte abundance; negative values indicate attenuation. **(F)** PROGENy multivariate linear-model pathway scores in ReactiveOligo_high and ReactiveOligo_low tumors, compared using two-sided Wilcoxon rank-sum tests; P values are displayed. Virtual perturbation results are hypothesis-generating predictions and require experimental validation. **(F)** compared seven ReactiveOligo_high and seven ReactiveOligo_low samples. For scTenifoldKnk, network construction used 10 subsampled networks per perturbation, with 500 cells per network for Tumor_FN1_KO, Pericyte_FN1_KO, and Mature_CD44_KO and 400 cells per network for Reactive_CD44_KO.

We then used scTenifoldKnk to prioritize the candidate axis through virtual perturbation in four settings: Tumor_FN1_KO, Pericyte_FN1_KO, Reactive_CD44_KO, and Mature_CD44_KO. Tumor_FN1_KO yielded the largest predicted transcriptional response (88 significantly perturbed genes), followed by Mature_CD44_KO (36), Reactive_CD44_KO (22), and Pericyte_FN1_KO (3) (**Figure 7A**). Module-enrichment analysis most consistently implicated the ECM/Niche program; broad enrichment of Myelination, OPC-related, or Reactive/Stress modules was not observed at the same level (**Figure 7B**). Gene-level summaries showed predicted stress/cytoskeletal and oligodendrocyte-state changes after CD44 perturbation and broader niche-related rewiring after tumor FN1 perturbation (**Figures 7C, D**). Under the indirect model, reduced FN1-associated reactive oligodendrocyte abundance was predicted to attenuate hypoxia, EGFR, TGF-beta, and TNF-alpha-related tumor signaling, with smaller predicted effects on PI3K, WNT, MAPK, and NF-kappaB programs (**Figure 7E**). Tumors in ReactiveOligo_high contexts showed higher TGF-beta activity and a borderline higher EGFR signal, with concordant directional trends in additional pathways (**Figure 7F**). These computational results prioritize tumor-derived FN1 as a candidate niche-organizing signal but do not substitute for experimental perturbation.

## Discussion

In this study, bidirectional MR identified subtype-specific genetic associations between lung cancer liability and brain structural or functional traits. SCLC showed the most coherent white-matter-related pattern, involving the superior cerebellar peduncle and cerebellar-related functional connectivity, whereas LUSC was more strongly associated with cortical traits in frontal, parietal, and sensorimotor regions. LUAD showed only limited exploratory functional associations without consistent structural support. Importantly, MR estimates effects of inherited genetic liability and do not demonstrate that an existing tumor actively remodels the brain before metastasis. The imaging findings instead provided a hypothesis-generating framework for identifying biological programs that might be relevant to the interaction between SCLC and the brain microenvironment. This focus is biologically plausible given the marked neuroendocrine plasticity and strong brain tropism of SCLC (61, 62).

The SCLC-associated white-matter pattern motivated our focus on oligodendrocytes because these cells are the principal regulators of myelin formation, axonal insulation, and white-matter homeostasis. Single-cell analysis identified mature-like, intermediate, and reactive-like oligodendrocyte states, consistent with the recognized transcriptional heterogeneity of this lineage (63). Reactive-like cells showed attenuation of canonical myelination programs together with enrichment of immediate-early and stress-response genes, whereas mature-like cells retained MOBP, PLP1, MBP, CNP, and MOG. These features are consistent with a stress-adapted, myelin-remodeling transcriptional state but do not by themselves demonstrate histological demyelination. Persistent fibronectin deposition can impair oligodendrocyte differentiation and remyelination, and integrin signaling contributes to oligodendrocyte survival and maturation (64–67). Nevertheless, white-matter alterations are multicellular phenomena involving astrocytes, microglia, endothelial cells, and extracellular-matrix components (39). Our oligodendrocyte-centered interpretation is therefore a biologically motivated, non-exclusive hypothesis.

Integrating NicheNet and CellChat prioritized a predicted FN1-associated tumor-oligodendrocyte interaction program, with neuronal-like malignant SCLC cells as the major inferred sender population. FN1 was emphasized over other ligands as a convergent candidate because it was retained within the displayed high-priority NicheNet ligand set, was broadly expressed across malignant states, and received additional, although exploratory, support from external spatial and virtual perturbation analyses. FN1 is a key extracellular-matrix component with established roles in matrix organization, invasion, and metastatic niche biology (68). Previous SCLC brain-metastasis studies have highlighted astrocyte-mediated support, developmental mimicry, tumor plasticity, and immunosuppressive programs (61, 62). Our findings extend this literature by suggesting that oligodendrocyte-associated white-matter biology may represent an additional component of the metastatic microenvironment. In the proposed model, FN1 may cooperate with CD44 and integrin-containing receptor complexes, while other extracellular-matrix ligands such as TGFBI, TNC, and laminin-related components may provide signaling redundancy. Alpha-V/integrin signaling is linked to extracellular-matrix sensing, TGF-beta activation, epithelial-mesenchymal plasticity, angiogenesis, and metastasis (69–72). However, these ligand-receptor relationships remain computational predictions and require direct functional testing.

Cross-dataset and virtual perturbation analyses refined this indirect model without establishing it as a mechanism. In independent NSCLC brain metastasis data, upper-tail rather than mean tumor FN1 better tracked reactive-state features, suggesting that localized FN1-high subpopulations may be more informative than global abundance. Spatial analyses similarly suggested regional proximity between FN1-enriched tumor territories and reactive-like oligodendrocyte signals. Virtual tumor FN1 knockout produced the largest predicted response among the tested perturbations and primarily affected ECM/Niche organization, while pathway summaries linked FN1-associated reactive oligodendrocyte abundance to TGF-beta/EGFR-related and hypoxia/inflammatory tumor programs. Because the correlation evidence was borderline, the spatial findings indicate co-localization, and the knockout was entirely in silico, these observations should be regarded as convergent hypothesis-generating evidence rather than definitive validation. Clinically, the model does not yet support patient stratification or therapeutic intervention. Its prognostic and therapeutic relevance will require prospective cohorts, longitudinal imaging, and experimental models (73).

Several limitations should be acknowledged. First, the GWAS datasets were derived mainly from European populations, and replication in ancestrally diverse cohorts is needed. Second, MR evaluates inherited genetic liability and cannot capture dynamic tumor-induced processes during metastatic evolution. The exploratory fMRI findings, particularly for LUAD and reverse MR, are also sensitive to physiological and technical variability and multiple testing. Third, the single-cell and spatial datasets were generated from established brain metastases rather than pre-metastatic tissue; consequently, they provide biological context but cannot bridge the MR findings directly to an early metastatic mechanism. Fourth, integrating public datasets across cancer histologies, cohorts, and experimental platforms introduces batch effects and biological heterogeneity that may influence cross-study comparisons. Fifth, NicheNet, CellChat, CellTrek proximity, and scTenifoldKnk analyses are computational and cannot demonstrate functional signaling. The absence of publicly available GWAS summary statistics for brain metastasis also precluded a direct MR analysis of metastatic risk. Future work should combine matched primary and metastatic specimens, longitudinal patient-level imaging, spatial proteomics or multiplex imaging, and oligodendrocyte-tumor co-culture or organoid perturbation to test the proposed FN1-associated model.

## Conclusions

In summary, this study identifies subtype-specific genetic associations between lung cancer liability and brain imaging phenotypes, with SCLC showing the clearest white-matter-related pattern. Integrated single-cell, cross-dataset, spatial, and virtual perturbation analyses suggest an indirect FN1-associated tumor-oligodendrocyte model linked to reactive oligodendrocyte transcriptional states. The findings provide a hypothesis-generating framework for future experimental and clinical investigation rather than evidence of a proven pre-metastatic or therapeutic mechanism.

## Data Availability

The original contributions presented in the study are included in the article/**Supplementary Material**. Further inquiries can be directed to the corresponding author.

## Supplementary material

Supplementary Figure 1 Suggestive MR associations of SCLC liability with resting-state fMRI traits. **(A)** Forest plot of IVW associations meeting the prespecified exploratory threshold of nominal P < 0.01. Effect estimates and 95% confidence intervals represent changes in standardized functional MRI traits associated with genetically predicted SCLC liability. **(B)** Network representation of the corresponding functional connections; edge color denotes effect size. These findings were exploratory and were not corrected for the full number of fMRI traits.

Supplementary Figure 2 Suggestive MR associations of LUSC liability with resting-state fMRI traits. **(A)** Forest plot of IVW associations meeting the prespecified exploratory threshold of nominal P < 0.01. Effect estimates and 95% confidence intervals are shown. Heter, Pleio, and MRPRESSO denote P values from Cochran’s Q, MR-Egger intercept, and MR-PRESSO global tests, respectively. **(B)** Network representation of corresponding connectivity and node-amplitude traits. Node color denotes the effect on spontaneous activity, gray denotes no mapped node effect, and edge color denotes the connectivity effect. These results were exploratory and not multiplicity-confirmed.

Supplementary Figure 3 Suggestive MR associations of LUAD liability with resting-state fMRI traits. **(A)** Forest plot of IVW associations meeting the prespecified exploratory threshold of nominal P < 0.01, with effect estimates and 95% confidence intervals. Heter, Pleio, and MRPRESSO denote P values from Cochran’s Q, MR-Egger intercept, and MR-PRESSO global tests, respectively. **(B)** Network representation of corresponding connectivity and node-amplitude traits. Node and edge colors denote effect sizes. These results were exploratory and not supported by statistically significant structural MRI associations.

Supplementary Figure 4 Exploratory reverse MR associations of resting-state fMRI traits with lung cancer liability. **(A)** Forest plots of IVW associations meeting nominal P < 0.01, with effect estimates and 95% confidence intervals. Heter, Pleio, and MRPRESSO denote P values from Cochran’s Q, MR-Egger intercept, and MR-PRESSO global tests, respectively. **(B)** Network representation of functional connections associated with SCLC or LUSC liability; edge color denotes effect size. Reverse associations were considered hypothesis generating.

Supplementary Figure 5 Quality control and annotation details of the public single-cell dataset. **(A)** Violin plots showing the distributions of quality-control metrics across samples or clusters, including the number of detected features per cell (nFeature_RNA), total UMI counts per cell (nCount_RNA), and the proportion of mitochondrial transcripts (percent.mt). **(B)** Doublet classification of the public single-cell dataset. Potential doublets were identified using a computational doublet-detection approach, and cells were classified as singlets or doublets prior to downstream analyses. **(C)** Reference-based and marker-assisted annotation of major cell populations. Cell identities were assigned by integrating reference-based annotation with canonical marker gene expression, thereby supporting the classification of major cell types in the SCLC brain metastasis microenvironment. **(D)** Heatmap showing representative cluster marker genes across cell clusters. Top marker genes for each cluster are displayed to support cluster annotation and the identification of major malignant and non-malignant cell populations.

Supplementary Figure 6 copyKAT-based validation and selection of high-confidence cell populations. **(A)** UMAP showing copyKAT-based classification of the public SCLC brain metastasis single-cell dataset. Cells were classified as aneuploid, diploid, or not defined on the basis of inferred large-scale copy-number variation patterns. **(B)** Distribution of copyKAT predictions across clusters. The proportions of aneuploid, diploid, and not defined cells are shown for each cluster, illustrating the relative enrichment of malignant and non-malignant populations across transcriptionally defined clusters. **(C)** Selection logic for high-confidence cell populations used in downstream analyses. Strict tumor cells were defined as cells annotated as tumor and classified as aneuploid by copyKAT, whereas strict oligodendrocyte-like cells were defined as oligodendrocyte-like cells classified as diploid by copyKAT.

Supplementary Figure 7 Oligodendrocyte state characterization. **(A)** Violin plots showing module-score-based characterization of oligodendrocyte-like cell states. The distributions of mature/myelinating, oligodendrocyte precursor-like, and reactive/stress-associated transcriptional programs are shown across Mature_like, Oligo_intermediate, and Reactive_high oligodendrocyte subgroups. **(B)** Violin plots showing receiver-side receptor expression across oligodendrocyte-like states. Expression of CD44, ITGAV, ITGB1, and ITGB8 is compared among Mature_like, Oligo_intermediate, and Reactive_high oligodendrocyte subgroups. **(C)** Correlation plots showing the relationships between receptor expression and oligodendrocyte state scores. Spearman correlation analyses were performed for ITGB1 and CD44 against reactive and mature oligodendrocyte scores. Each dot represents one oligodendrocyte-like cell, colored by oligodendrocyte subgroup. The fitted line indicates the overall trend of association.

Supplementary Figure 8 Full NicheNet and CellChat results. **(A)** Full NicheNet ligand-activity ranking. Candidate tumor-derived ligands were ranked by corrected AUPR, with FN1 highlighted. **(B)** Predicted extracellular-matrix-receptor ligand-receptor interactions inferred by CellChat between malignant SCLC states and oligodendrocyte-like subgroups. **(C)** Predicted secreted-signaling interactions inferred by CellChat. In **(B, C)**, each bubble represents one computationally inferred pair; size and color encode predicted communication probability. These analyses do not establish direct functional signaling.

Supplementary Figure 9 External evaluation in GSE223499 NSCLC brain metastasis samples. **(A)** UMAP showing refined broad annotation of brain metastasis samples from GSE223499, including tumor-like, pericyte-like, endothelial, microglial, T/NK, astrocyte-like, and oligodendrocyte-like populations. **(B)** UMAP showing oligodendrocyte-like cell states resolved in GSE223499, including Mature_like, Reactive_like, and OPC_like states. **(C)** Violin plots showing module scores for mature/myelinating, reactive/stress-associated, and OPC-like transcriptional programs across oligodendrocyte-like cell states. **(D)** Dot plot showing representative marker genes across oligodendrocyte-like states. Mature-like cells preferentially expressed myelination-associated genes, reactive-like cells expressed stress-response genes, and OPC-like cells expressed precursor-associated genes. **(E)** Supportive ECM-associated background in GSE223499. Left, composition of FN1-high cells across broad cell types. Right, expression of candidate receptor genes (CD44, ITGB1, ITGAV, and ITGB8) across oligodendrocyte-like states.
